# RETRACTION: Effects of Rapid Rehabilitation Nursing on Surgical‐Site Wound Infection and Postoperative Complications of Patients Undergoing Thoracoscopic Lung Cancer Surgery: A Meta‐Analysis

**DOI:** 10.1111/iwj.70271

**Published:** 2025-03-03

## Abstract

**RETRACTION**: LiY. , HuangD. , MeiC. , XieN. , and LiL. , “Effects of Rapid Rehabilitation Nursing on Surgical‐Site Wound Infection and Postoperative Complications of Patients Undergoing Thoracoscopic Lung Cancer Surgery: A Meta‐Analysis,” International Wound Journal 21, no. 2 (2024): e14701, 10.1111/iwj.14701.PMC1083192142052992

The above article, published online on 01 February 2024, in Wiley Online Library (http://onlinelibrary.wiley.com/), has been retracted by agreement between the journal Editor in Chief, Professor Keith Harding; and John Wiley & Sons Ltd. Following an investigation by the publisher, all parties have concluded that this article was accepted solely on the basis of a compromised peer review process. The editors have therefore decided to retract the article. The authors did not respond to our notice regarding the retraction.

**RETRACTION**: Y. Li , D. Huang , C. Mei , N. Xie , and L. Li , “Effects of Rapid Rehabilitation Nursing on Surgical‐Site Wound Infection and Postoperative Complications of Patients Undergoing Thoracoscopic Lung Cancer Surgery: A Meta‐Analysis,” International Wound Journal 21, no. 2 (2024): e14701, 10.1111/iwj.14701.PMC1083192142052992

The above article, published online on 01 February 2024, in Wiley Online Library (http://onlinelibrary.wiley.com/), has been retracted by agreement between the journal Editor in Chief, Professor Keith Harding; and John Wiley & Sons Ltd. Following an investigation by the publisher, all parties have concluded that this article was accepted solely on the basis of a compromised peer review process. The editors have therefore decided to retract the article. The authors did not respond to our notice regarding the retraction.

